# Nuclear Factor of Activated T Cells Is Activated in the Endothelium of Retinal Microvessels in Diabetic Mice

**DOI:** 10.1155/2015/428473

**Published:** 2015-03-31

**Authors:** Anna V. Zetterqvist, Fabiana Blanco, Jenny Öhman, Olga Kotova, Lisa M. Berglund, Sergio de Frutos Garcia, Raed Al-Naemi, Maria Wigren, Paul G. McGuire, Laura V. Gonzalez Bosc, Maria F. Gomez

**Affiliations:** ^1^Department of Clinical Sciences in Malmö, Lund University, 20502 Malmö, Sweden; ^2^Departamento de Biofísica, Facultad de Medicina, Universidad de la República, 11800 Montevideo, Uruguay; ^3^Department of Cell Biology and Physiology, University of New Mexico Health Sciences Center, Albuquerque, NM 87131, USA

## Abstract

The pathogenesis of diabetic retinopathy (DR) remains unclear but hyperglycemia is an established risk factor. Endothelial dysfunction and changes in Ca^2+^ signaling have been shown to precede the onset of DR. We recently demonstrated that high extracellular glucose activates the Ca^2+^/calcineurin-dependent transcription factor NFAT in cerebral arteries and aorta, promoting the expression of inflammatory markers. Here we show, using confocal immunofluorescence, that NFAT is expressed in the endothelium of retinal microvessels and is readily activated by high glucose. This was inhibited by the NFAT blocker A-285222 as well as by the ectonucleotidase apyrase, suggesting a mechanism involving the release of extracellular nucleotides. Acute hyperglycemia induced by an IP-GTT (intraperitoneal glucose tolerance test) resulted in increased NFATc3 nuclear accumulation and NFAT-dependent transcriptional activity in retinal vessels of NFAT-luciferase reporter mice. In both Akita (*Ins2^+/−^*) and streptozotocin- (STZ-) induced diabetic mice, NFAT transcriptional activity was elevated in retinal vessels. *In vivo* inhibition of NFAT with A-285222 decreased the expression of *OPN* and *ICAM-1* mRNA in retinal vessels, prevented a diabetes driven downregulation of anti-inflammatory IL-10 in retina, and abrogated the increased vascular permeability observed in diabetic mice. Results identify NFAT signaling as a putative target for treatment of microvascular complications in diabetes.

## 1. Introduction

Diabetic retinopathy (DR) is still one of the leading causes of vision loss worldwide. Even though the underlying pathogenesis is not clear, hyperglycemia is an important risk factor [1]. We have recently demonstrated that modest elevations of extracellular glucose activate the Ca^2+^/calcineurin-dependent transcription factor NFAT (nuclear factor of activated T cells) in smooth muscle cells of conduit and resistance arteries [2, 3]. The effect of glucose involved the local release of extracellular nucleotides, such as ATP and UTP, acting on P2Y receptors, leading to increased intracellular Ca^2+^ ([Ca^2+^]_i_) and subsequent activation of calcineurin and NFAT [2]. ATP and UTP are vasoactive signals able to increase [Ca^2+^]_i_ in the retina, via stimulation of purinergic receptors including P2Y4 [4]. Also, high glucose has been shown to increase extracellular ATP in rat retinal cell cultures [5]. Therefore, we hypothesize that hyperglycemia may activate NFAT in retinal microvessels.

Inflammation and endothelial activation are important early steps in the development of DR, leading to leukostasis, platelet activation, and upregulation of inflammatory cytokines [6]. The NFAT family (NFATc1–c4) plays a central role in the production of cytokines in immune cells and in the regulation of T-cell proliferation. We and others have shown that in conduit and resistance arteries and in cultured vascular cells NFAT regulates the expression of inflammatory genes, such as IL-6, allograft inflammatory factor 1 (AIF-1), tissue factor (TF), cyclooxygenase 2 (Cox-2), and osteopontin (OPN) [3, 7–9]. Expression of endothelial activation markers, such as VCAM-1 and E-selectin, is also dependent on NFAT signaling in cultured smooth muscle and endothelial cells, respectively [10, 11]. More recently, we showed that* in vivo* inhibition of NFAT signaling reduces* ICAM-1* mRNA expression in the aortas of diabetic Apoe^−/−^ mice [9], a leukocyte adhesion molecule that is elevated in retinal vessels from diabetic mice and patients [6, 12].

Another early feature of DR is the breakdown of the blood-retinal barrier (BRB) [13], which results in vascular leakage and development of retinal edema. Earlier investigations focused on vascular endothelial growth factor (VEGF), shown to induce rapid phosphorylation of tight junction proteins and increased retinal permeability [14]. However, recent* in vivo* kinetic data show that the retinal barrier function is compromised before VEGF levels are increased and use of a neutralizing anti-VEGF antibody is not effective at reducing permeability at early stages of diabetes (8 weeks) [15]. In the context of angiogenesis [16, 17], VEGF appears to be an upstream activator of NFAT, but both VEGF and its receptor VEGFR2 are also downstream targets of NFAT in endothelial cells [18, 19]. Hence, a role of NFAT in the early changes of DR cannot be ruled out.

Here, we investigated the effects of high glucose and diabetes on NFAT activation in a streptozotocin (STZ) model of diabetes and in hyperglycemic Akita (*Ins2* ^+/−^) mice. We also explored the effects of* in vivo* NFAT-signaling inhibition on the expression of inflammatory mediators, endothelial adhesion molecules, and vascular permeability in diabetic mice.

## 2. Research Design and Methods

### 2.1. Animals

All animal protocols in this study were reviewed and approved by Institutional Animal Care and Use Committees, University of New Mexico, School of Medicine and Lund University, Sweden. The following mice strains (number of animals per strain indicated) were bred in our animal facilities: FVBN 9x-NFAT-luciferase reporter (NFAT-luc [2, 7, 20]; *N* = 133), Akita (*Ins2* ^+/−^), and wild-type (*Ins2* ^+/+^) littermates (stock number 003548, C57Bl/6J background, Jackson Laboratories, Maine, here referred to as Akita and WT; *N* = 31). We also generated Akita/NFAT-luc mice and WT/NFAT-luc littermates (*N* = 43), which were backcrossed at least four generations into the C57Bl/6J background. Wild-type adult BALB/c (*N* = 76), C57Bl/6 (Taconic, Europe; *N* = 12), and ApoE^−/−^ (B6.129P2-Apoe^*tm1Unc*^/J; Charles River, Sulzfeld, Germany; *N* = 22) mice were also used. Animals had free access to tap water and were fed normal chow diet. Retinas, cerebral arteries, aortas, and plasma were used. Both eyes were enucleated from all mice included in this study; however, not always both eyes were used (some were stored for future studies). If both eyes were used, each eye was processed differently for the various types of measurements detailed below. For* in vivo* experiments using A-285222, the drug was administered intraperitoneally (i.p.) once a day for the duration of the experiments, at 0.15–0.29 mg/kg body weight depending on the mouse strain and based on previous studies [3]. A-285222 is a low molecular weight (461 daltons), cell permeable organic compound that inhibits all NFAT family members and was provided by Abbott Laboratories (Abbott Park, IL). For experiments involving collection of blood at termination, mice were anaesthetized by i.p. injection of 7.5 mg ketamine hydrochloride and 2.5 mg xylazine per 100 mg body weight and euthanized by exsanguination through cardiac puncture. For all other experiments, mice were euthanized by cervical dislocation or pentobarbital injection (200 mg/kg) followed by decapitation.

### 2.2. Isolation of Retinal Vessels

Eyes were enucleated and retinas dissected in ice-cold Ca^2+^-free physiological saline solution (PSS; in mmol/L: NaCl, 135; KCl, 5.9; MgCl_2_, 1.2; Hepes, 11.6; glucose, 2.0; pH 7.4). Retinal vessels were isolated from one eye as previously described [21]; the second eye was used for measurements in whole retina (see below). A total of 194 eyes were used for isolation of retinal vessels. Vessel integrity after isolation was confirmed by staining the vessel networks with smooth muscle *α*-actin antibody and the fluorescent nucleic acid dye SYTOX green (1 : 3000, Molecular Probes, Invitrogen, Paisley, UK). Intact retinal endothelial and smooth muscle cells were visualized by confocal microscopy as described below. Potential residual levels of neuronal and glial contamination were assessed by quantitative RT-PCR measuring* Tau* and* GFAP* expression, respectively, using* 18S* as endogenous control.

### 2.3. RNA Isolation, Conventional RT-PCR, and Quantitative Real Time PCR

Total RNA was extracted from whole retinas, isolated retinal vessels, and aortas from mice and from human retinal microvessel endothelial cells (HRMVECs) as previously described [3, 21]. HRMVECs (catalog number ACBRI 181) were purchased from Cell Systems (Kirkland, WA) and grown in Medium 131 containing microvascular growth supplement (Gibco, Life Technologies Corp., Carlsbad, CA), 50 U/mL penicillin, and 50 *μ*g/mL streptomycin at 37°C, 95% air, and 5% CO_2_. For conventional RT-PCR, RNA extraction was followed by reverse transcription using RevertAid (Fermentas GMBH, St. Leon-Rot, Germany) and oligo-dT primers. cDNA was amplified (HotStarTaq Master Mix Kit, Qiagen) using NFAT isoform specific primers as previously described [7]. PCR products were separated by agarose gel electrophoresis and confirmed by sequencing. Equal amounts of RNA for whole retina and isolated retinal vessel preparations were used for the reverse transcription reactions. For quantitative real time PCR, cDNA was synthesized using random hexamer primers and amplified on a 7900HT TaqMan system (Applied Biosystems, Carlsbad, CA) using TaqMan Gene Expression assays for* Tau* (Mm00521988_m1),* GFAP* (Mm01253033_m1),* OPN* (Mm00436767_m1),* ICAM-1* (Mm00516023_m1) with* 18S* (Hs999999021_s1),* HPRT* (Mm00446968_m1),* Cyclophilin B* (Mm00478295_m), and* GAPDH* (4352339E) as endogenous controls. Relative quantities of target genes were calculated using the comparative threshold method (ΔΔC_*t*_) and experiments were performed in triplicate.

### 2.4. Intraperitoneal Glucose Tolerance Test (IP-GTT)

BALB/c mice fasted for 16 hours followed by i.p. injection of glucose (2 g/kg body weight) or saline (vehicle). Blood glucose was measured in whole venous blood from the tail vein (OneTouch glucometer, LifeScan, Inc., CA). Mice were euthanized 30 minutes or 6 hours after the glucose bolus, after which eyes were enucleated and retinas were dissected free and used for measurements of NFATc3 nuclear accumulation by confocal immunofluorescence microscopy or for measurements of luciferase activity as explained below, respectively.

### 2.5. Streptozotocin (STZ) Treatment

Mice were injected i.p. with STZ (60 mg/kg body weight in citrate buffer, pH 4.5) or vehicle (citrate buffer), once a day during 4 or 6 days, for NFAT-luc and BALB/c, respectively, as previously described [3]. Mice were weighed and glucose levels measured at various time points. NFAT-luc mice (control, diabetic, and diabetic treated with the NFAT blocker A-285222) were euthanized at different time points as specified in the results. BALB/c mice (control and diabetic with or without A-285222, resp.) were euthanized 4 weeks after the first STZ/vehicle injections.

### 2.6. Confocal Immunofluorescence

For measurements of NFATc3 nuclear accumulation, retinas and cerebral arteries from BALB/c mice were dissected in ice-cold Ca^2+^-free PSS and treated* ex vivo* as described in Results section. Alternatively, retinas were fixed directly after IP-GTT. Whole retinas were fixed with Histochoice MB (AMRESCO Inc., Solon, OH), blocked and permeabilized with 2% BSA containing 0.2% Triton X-100 in PBS for 2 hours, and incubated with rabbit polyclonal anti-NFATc3 (1 : 100, Santa Cruz Biotechnology, Santa Cruz, CA) overnight at 4°C and with Cy5-anti-rabbit IgG (1 : 500, Jackson ImmunoResearch, West Grove, PA) for 2 hours at room temperature. Human umbilical vein endothelial cells (HUVEC; Gibco, Life Technologies Corp., Carlsbad, CA) and HRMVECs were grown on glass coverslips and stained with goat polyclonal anti-NFATc2 (Santa Cruz Biotechnology, Santa Cruz, CA) and with Cy5-anti-goat IgG (1 : 500, Jackson ImmunoResearch, West Grove, PA). Nuclei were stained with SYTOX Green. Cerebral arteries were processed as previously described [2, 3]. NFATc3 or NFATc2 and nuclear regions were detected by monitoring Cy5 and green fluorescence, respectively, on a Zeiss LSM 5 laser scanning confocal microscope. Images were obtained at 63x magnification and mean fluorescence intensity of nuclear NFATc3 or NFATc2 was quantified using the Zeiss LSM 5 analysis software as previously described [3, 7]. Orientation of the nuclei was used to distinguish endothelial from smooth muscle cells. For visualization of colocalized image regions or double tagged regions (red: NFATc3 tagged with Cy5 and green: nuclear regions tagged with SYTOX Green), the crosshair function of the LSM program was used. This tool leads to the distribution of all image pixels over 4 quadrants in a scattergram according to their intensity levels, with the background pixels sorted into the bottom left quadrant, the single-tagged pixels (either red or green) into the upper left and bottom right quadrants, and the pixels having an intensity above the background in both channels (i.e., colocalized pixels) represented by the upper right quadrant. The image pixels corresponding to the upper right quadrant are then color-coded white in the original image to allow fast identification of colocalized areas. Also, staining of retinal whole-mounts with antibodies against von Willebrand factor (1 : 400, DAKO), smooth muscle *α*-actin (1 : 400, Sigma), and platelet-derived growth factor *β*-receptor (PDGFR*β*, 1 : 100, Santa Cruz) was performed to identify endothelial, smooth muscle cells and pericytes, respectively (see Supplementary Figure 1 in the Supplementary Material available online at http://dx.doi.org/10.1155/2015/428473). For quantification, multiple fields for each retina, vessels, or coverslips were imaged and analyzed under blind conditions.

### 2.7. Luciferase Reporter Assay

Luciferase activity was measured in intact retinas and in isolated retinal vessels as previously described [2, 7]; optical density was measured using a Tecan Infinite M200 instrument (Tecan Nordic AB, Mölndal, Sweden) and data was expressed as relative light units per microgram protein.

### 2.8. Cytokines in Plasma and Retina Homogenates

Cytokine concentrations were measured in plasma and retina homogenates using an inflammation 7-Plex kit (Meso Scale Discovery, Rockville, MD). The lower detection limits were within the range specified by the manufacturer. Plasma OPN was assayed using Quantikine Mouse OPN ELISA kit (R&D Systems, Abingdon, UK). Absorbance was measured at 450 nm and the lower limit of detection was 5.7 pg/mL. Plasma from OPN^−/−^ mice [22] (kindly provided by Anna Hultgårdh, Lund University) was used as negative control.

### 2.9. Quantitative Assessment of Blood-Retinal Barrier Permeability

FITC-labelled albumin (45 mg/kg; Sigma) was administered i.v. and 1 hour later, mice were anesthetized, and a blood sample was collected from the left ventricle. Mice were perfused with PBS and euthanized by cardiac puncture. The fluorescence of retinal extracts and a 1 : 1000 dilution of plasma were measured at 485 nm (Ex) and 515 nm (Em). The concentration of FITC-albumin was calculated from a standard curve of FITC-labelled albumin in PBS and the blood-retinal barrier permeability was calculated as follows and was expressed as (*μ*L/mg∗hr/eye):

(1) FITC−albumin  retina  (mg)/Retina  dry  weight  (mg)[FITC−albumin]  (mg)/Plasma  (ul)×Circulation  time  (h).

### 2.10. Statistics

Results are expressed as means ± SEM unless otherwise specified. Statistical analysis was performed using GraphPad software (Prism 5.01). The use of parametric or nonparametric tests was based on results from analyses of distributions. Statistical significance was determined using Student's *t*-test; Kruskal-Wallis followed by Dunn's multiple comparison test for nonparametric data; or one- or two-way ANOVA as stated in the figure legends, followed by Bonferroni post hoc tests (^∗^ *P* < 0.05, ^∗∗^ *P* < 0.01, and ^∗∗∗^ *P* < 0.001). Pearson's test was used for correlation analyses.

## 3. Results

### 3.1. Expression of NFATc Transcription Factors in Mouse Retina

**Figure 1:**
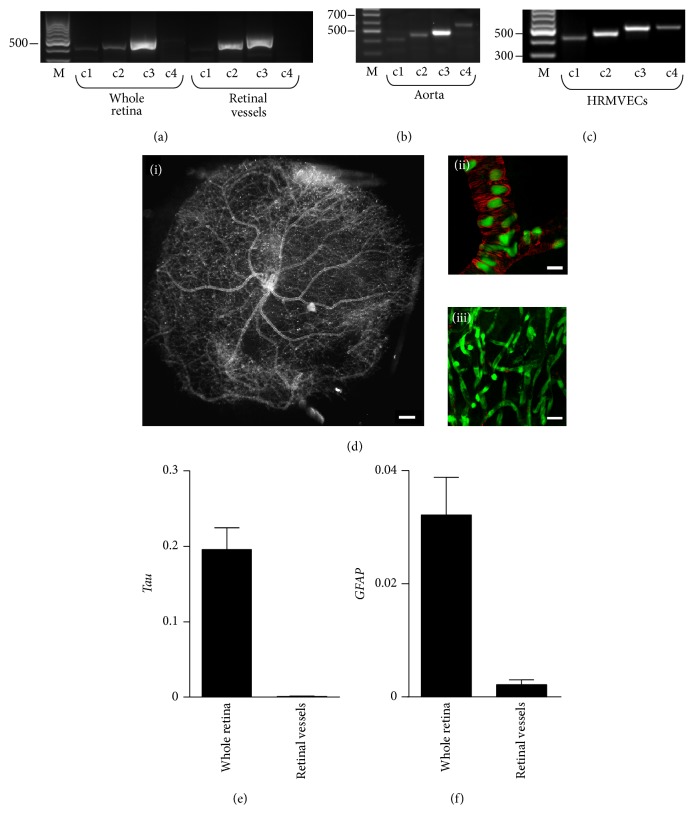
Expression of NFATc transcription factor genes in whole retina and in retinal microvessels. (a) RT-PCR analysis of NFAT isoform (c1–c4) expression in whole retinas and isolated retinal vessels from nondiabetic FVBN mice (*N* = 8). ((b)-(c)) All NFAT family members are expressed in mouse aorta (b) and in cultured human retinal microvascular endothelial cells (HRMVECs, (c)). Markers (M) for 300, 500, and 700 bp are indicated. (d)(i) Dark field/side illumination of a vascular network isolated from whole retina as described in material and methods (Section 2.2). (d)(ii) Confocal image showing smooth muscle *α*-actin positive staining (red) and nuclei (green) of an isolated retinal vessel preparation. Note the lack of surrounding nonvascular tissue (black background). (d)(iii) Confocal image of a smaller caliber vessel network stained as in (d)(ii) showing the absence of smooth muscle *α*-actin positive cells. Scale bars = 500 *μ*m for (d)(i); 20 *μ*m for (d)(ii)-(d)(iii). (e)-(f) Quantitative RT-PCR data showing expression levels of neuronal maker Tau (e) and glial marker GFAP (f) in whole retina and in isolated retinal vessels, both normalized to 18S. *N* = 6 retinas/group.

RT-PCR was used to examine the expression of NFATc transcription factors in mouse retina.* NFATc2* and* NFATc3* were readily detected in whole retina from mouse, whereas expression of* NFATc1* was very low and of* NFATc4* very low to undetectable (Figure 1(a)). Using the same primer pairs as for retina, all four NFAT family members were detected in intact mouse aorta (Figure 1(b)) and in cultured human retinal microvascular endothelial cells (HRMVECs; Figure 1(c)). In intact mouse retinal vessels (Figure 1(d)), virtually devoid of neuronal and glial cells (Figures 1(e)-1(f)), both* NFATc2* and* NFATc3* were detected (Figure 1(a)). Although the level of expression is not a determinant of transcription factor activity, we chose to focus on NFATc3, given that we previously established that this family member is glucose sensitive and activated in aorta and cerebral arteries in diabetic mice [2, 3, 9].

### 3.2. Acute Hyperglycemia Increases NFATc3 Nuclear Accumulation and NFAT-Dependent Transcriptional Activity in Retinal Microvessels

**Figure 2:**
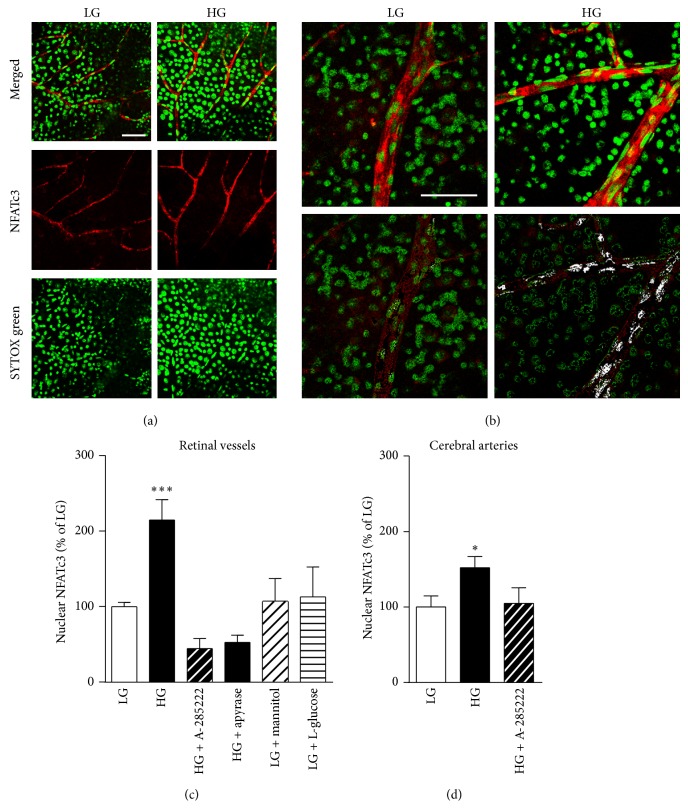
High glucose activates NFATc3 in the endothelium of retinal vessels. (a) Representative confocal immunofluorescence images of retinal whole-mounts, stimulated for 30 min in low (LG; 2 mmol/L; left panels) or high (HG; 20 mmol/L; right panels) extracellular glucose and stained for NFATc3 (red) and SYTOX green for identification of nuclei (green). Merged and individual channel images are shown. Endothelial cells were identified by the orientation of their nuclei. (b) Higher magnification confocal images of retinal whole-mounts stimulated and stained as in ((a), upper panels) and pseudocolored images to visualize colocalization of NFATc3 and SYTOX green (white; lower panels). Scale bars = 50 *μ*m. (c) Summarized mean fluorescence intensity data from experiments as in (a), showing NFATc3 nuclear accumulation after 30 min stimulation in LG or HG in the presence or absence of A-285222 (1 *μ*mol), or apyrase (3.6 U/mL), or after stimulation with LG plus mannitol (18 mmol/L), or LG plus L-glucose (18 mmol/L). *N* = 21 and 22 retinas for LG and HG, respectively; 5–7 retinas for the other stimulatory conditions. ^∗∗∗^ *P* < 0.001 versus all other groups. (d) Summarized mean fluorescence intensity data showing NFATc3 nuclear accumulation in mouse cerebral arteries after 30 min stimulation in LG or HG in the presence or absence of A-285222 (1 *μ*mol). *N* = 5/group. ^∗^ *P* < 0.05 versus LG.

Raising the extracellular glucose concentration* ex vivo* from 2 mmol/L to 20 mmol/L for 30 min significantly increased NFATc3 nuclear accumulation in the endothelium of retinal vessels (Figures 2(a)-2(b)). Exposure to high glucose results in a 115% increase in mean fluorescence intensity of nuclear NFATc3 when compared to vessels incubated under low glucose conditions (Figure 2(c)). This increase was prevented by the NFAT blocker A-285222 (1 *μ*mol) and also by the ectonucleotidase apyrase (3.6 U/mL), implicating extracellular nucleotides in the activation of NFATc3 (Supplementary Figures 2(a)-2(b)). Mannitol and L-glucose failed to induce NFATc3 nuclear accumulation, ruling out a possible osmotic effect of glucose and indicating that glucose needs to be metabolized in order to activate NFAT signaling (Supplementary Figures 2(c)-2(d)). For comparison, in Figure 2(d), a less dramatic NFATc3 activation is observed in the endothelium of mouse intact cerebral arteries after 30 min stimulation with HG, which was prevented by the NFAT blocker.

We have not been able to perform a quantitative analysis of NFATc2 activation in retinal whole-mounts due to substantial nonspecific binding of the antibodies tested, resulting in less reliable immunostaining. However, using HUVEC and HRMVECs, we have not seen any significant effect of glucose on NFATc2 nuclear accumulation (Supplementary Figure 3).

**Figure 3:**
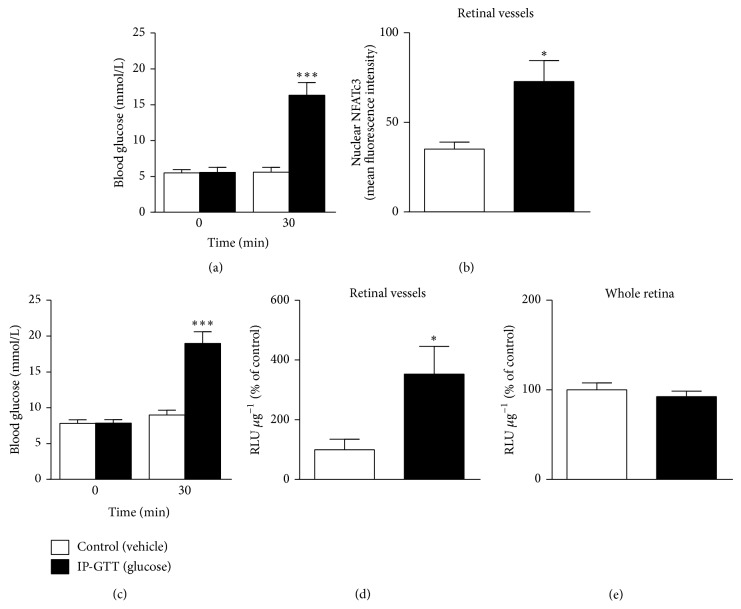
Acute hyperglycemia increases NFATc3 nuclear accumulation and NFAT-dependent transcriptional activity in retinal microvessels. (a) Blood glucose levels measured before and 30 min after an i.p. injection of glucose (2 g/kg; IP-GTT, black bars) or saline (control, white bars) in BALB/c mice, *N* = 7-8 mice/group. (b) Summarized data from confocal immunofluorescence experiments in the mice described in (a), showing increased NFATc3 nuclear accumulation in the retinal endothelium of hyperglycemic mice when compared to controls. (c) Blood glucose levels measured before and 30 min after an i.p. injection of glucose (2 g/kg; IP-GTT, black bars) or saline (control, white bars) NFAT-luc mice. (d)-(e) NFAT-dependent transcriptional activity in isolated retinal vessels (d) and whole retinas (e) in the NFAT-luc mice described in (c) measured 6 hours after i.p. injection of glucose or saline. Relative light units (RLU) in (d) and (e) were normalized to protein content and expressed as percentage of vehicle-treated control. *N* = 7–12 mice/group. ^∗^ *P* < 0.05 and ^∗∗∗^ *P* < 0.001 versus normoglycemic control group.

To test the effect of acute hyperglycemia on NFATc3 activation* in vivo*, i.p. glucose tolerance tests (IP-GTT) were performed and NFATc3 nuclear accumulation was determined in retinal vessels by confocal microscopy. Blood glucose levels were significantly higher in mice after the IP-GTT (Figure 3(a)), and this was accompanied by a 110% increase in mean fluorescence intensity of nuclear NFATc3 in the endothelium of mouse retinal microvessels (Figure 3(b)) compared to vehicle-injected mice. Levels of NFATc3 nuclear accumulation correlated with blood glucose concentrations (*r* = 0.600; *P* = 0.017). Nuclear accumulation of NFAT translated into increased NFAT-dependent transcriptional activity, as evidenced by significantly increased luciferase activity in isolated retinal vessels of transgenic NFAT-luc reporter mice after an IP-GTT (Figures 3(c)-3(d)). Interestingly, we could not detect any effect of acute hyperglycemia on NFAT-dependent transcriptional activity when measured in whole retina (Figure 3(e)), suggesting selective glucose-dependent activation of NFAT in the vascular retina.

### 3.3. Chronic Hyperglycemia Increases NFAT-Dependent Transcriptional Activity in Retinal Vessels in Two Different Models of Diabetes

**Figure 4:**
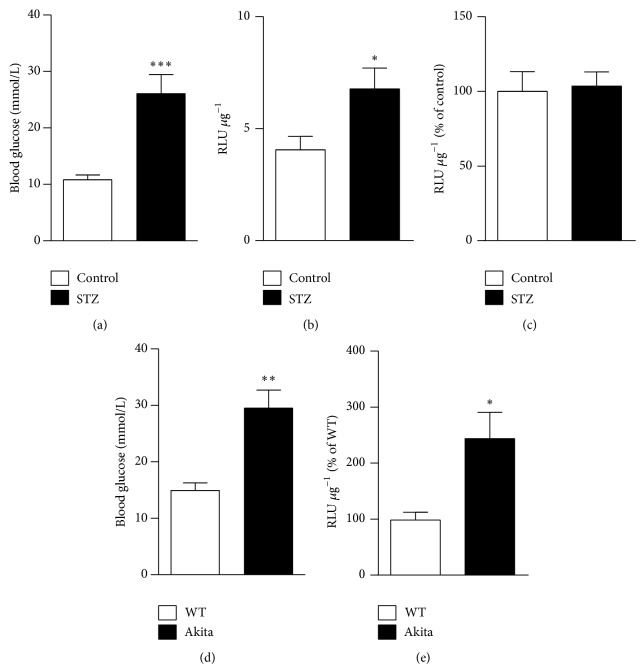
Chronic hyperglycemia increases NFAT-dependent transcriptional activity in retinal vessels from two different mouse models of diabetes. (a) Blood glucose levels in NFAT-luc mice, treated with STZ (60 mg/kg; black bars) or vehicle (citrate buffer; white bars), measured 12 days after the first injection. (b) NFAT-dependent transcriptional activity (RLU *μ*g^−1^) in isolated retinal vessels (*N* = 6-7 mice/group) and (c) in whole retinas (*N* = 4–7) from the mice in (a). Data is expressed as percentage of activity in normoglycemic control mice. (d) Blood glucose levels in Akita/NFAT-luc mice (black bars) and WT/NFAT-luc mice (white bars) at 6 weeks of age. (e) NFAT-dependent transcriptional activity (RLU *μ*g^−1^) in isolated retinal vessels from the same mice as in (d). Data is expressed as percentage of activity in WT control mice. *N* = 11–15 mice/group. ^∗^ *P* < 0.05; ^∗∗^ *P* < 0.01; and ^∗∗∗^ *P* < 0.001 versus corresponding normoglycemic control groups.

NFAT-luc mice were injected with STZ or vehicle once a day for 4 days and euthanized one week after the last injection (day 12). At this time point, blood glucose levels were significantly elevated in diabetic mice when compared to controls (Figure 4(a)). This resulted in a 68% increase in NFAT-dependent transcriptional activity in isolated retinal microvessels (Figure 4(b)), which correlated with blood glucose (*r* = 0.696, *P* = 0.037). NFAT-luciferase activity was also measured in whole retinas at day 12, but no differences were observed between diabetic and control mice (Figure 4(c)), not even at earlier or later time points (days 7 and 16; data not shown). Akita mice have a single amino acid substitution in the insulin 2 gene that causes misfolding of the insulin protein, progressive loss of *β*-cell function, and significant hyperglycemia as early as 4-5 weeks of age [23]. In our hands, at 6 weeks of age Akita/NFAT-luc mice had significantly increased blood glucose levels compared to WT/NFAT-luc mice (Figure 4(d)) and this was accompanied by a 148% increase in luciferase activity in isolated retinal microvessels (Figure 4(e)). Together, these results demonstrate activation of NFAT signaling in retinal vessels in two different models of type 1 diabetes.

### 3.4. Effect of NFAT-Signaling Inhibition on Inflammatory Cytokines and Endothelial Activation

**Figure 5:**
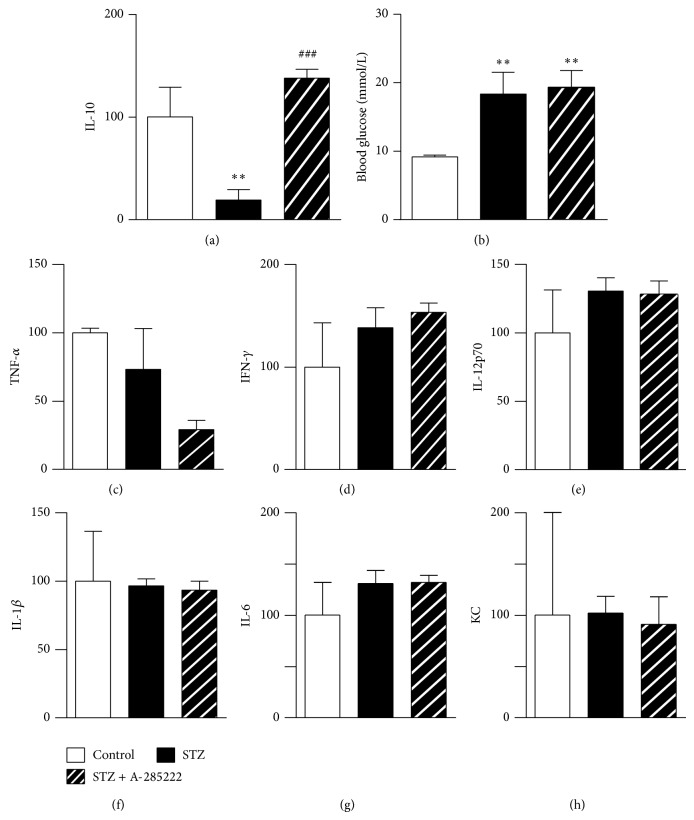
*In vivo* inhibition of NFAT restores levels of anti-inflammatory IL-10 in the retina of diabetic mice. (a) Levels of IL-10 in whole-retina homogenates from NFAT-luc mice, treated with STZ (60 mg/kg) or vehicle (citrate buffer). Mice received daily i.p. injections of A-285222 (0.15 mg/kg) or vehicle (saline) for two weeks (*N* = 2, 7, and 9 for control, STZ, and STZ + A-285222, resp.). ^∗∗^ *P* < 0.01 versus control and ^###^ *P* < 0.001 versus STZ. (b) Blood glucose levels in mice treated as in (a) (^∗∗^ *P* < 0.01 versus control; *N* = 7–12 mice/group). (c)–(h) TNF*α*, IFN-*γ*, IL-12p70, IL-1*β*, IL-6, and KC levels were determined in whole-retina homogenates from the same mice as in (a). Cytokines levels were normalized to protein content and are expressed as percentage of control. No significant differences were observed between the groups.

Nondiabetic (control) and STZ-induced diabetic NFAT-luc mice received daily i.p. injections of the NFAT blocker A-285222 (0.15 mg/kg body weight) or vehicle and were euthanized 16 days after the first injections. Diabetes resulted in significantly reduced levels of the anti-inflammatory cytokine IL-10 in whole-retina homogenates, which were completely restored in mice treated with A-285222 (Figure 5(a)). Blood glucose levels were significantly higher in STZ-treated mice when compared to control mice (Figure 5(b)) but were not affected by treatment with A-285222 (Figure 5(b)). The levels of other cytokines such as IFN-*γ*, IL-12p70, IL-1*β*, IL-6, GRO*α*/keratinocyte-derived chemokine (KC), and TNF*α* in whole-retina homogenates were not significantly affected by diabetes or treatment with A-285222 (Figures 5(c)–5(h)). When the same cytokines were examined in plasma, no differences were detected between diabetic mice treated with or without A-285222 (Table 1), suggesting that the effect on retinal IL-10 is local, rather than systemic.

**Figure 6:**
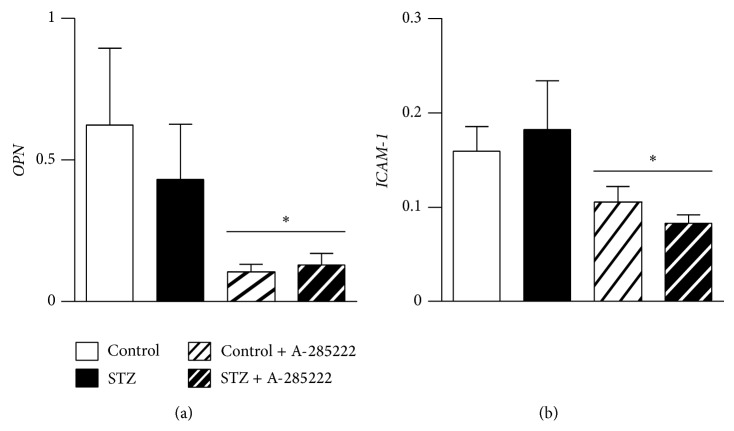
*In vivo* inhibition of NFAT reduces the expression of* OPN* and* ICAM-1* in retinal vessels. mRNA expression of (a) OPN and (b)* ICAM-1* in isolated retinal vessels from BALB/c mice treated with STZ (60 mg/kg) or vehicle (citrate buffer). Mice received for 4 weeks daily i.p. injections of A-285222 (0.29 mg/kg for 2 weeks followed by 0.15 mg/kg until termination) or vehicle (saline). Expression levels were determined by quantitative RT-PCR and* HPRT* was used as endogenous control. *N* = 7-8 mice/group. Two-way ANOVA revealed a significant effect of A-285222 on* OPN* and* ICAM-1* expression; ^∗^ *P* < 0.05.

In a separate set of experiments using BALB/c mice and contrary to expectations, one month after the first STZ injection we did not detect enhanced* OPN* or* ICAM-1* mRNA in the retinal vessels of diabetic mice (Figures 6(a)-6(b)). OPN levels in the aortas of these same diabetic mice were elevated when compared to controls [3], suggesting differential regulation of OPN expression depending on the vascular bed. Similar to what we observed in retinal vessels from BALB/c mice, neither* OPN* mRNA in retinal vessels (Supplementary Figure 4(a)) nor OPN protein levels in whole-retina homogenates (data not shown) were changed in NFAT-luc mice after 2 weeks of diabetes. Furthermore,* OPN* mRNA in retinal vessels of Akita mice was not changed (Supplementary Figure 4(b)). STZ-induced diabetes in hyperlipidemic* Apoe* ^−/−^ mice, on the other hand, resulted in a 2.1-fold increase in* OPN* mRNA in retinal vessels, 8 weeks after the first STZ injection (Supplementary Figure 4(c)), highlighting potential differences in the regulation of OPN depending on mice strain, duration of diabetes, and/or blood lipid levels. Despite the lack of diabetes-induced* OPN* and* ICAM-1* expression in BALB/c mice, daily injections with A-285222 lowered the levels of* OPN* and* ICAM-1* mRNA (Figures 6(a)-6(b)). No changes in plasma levels of OPN, IL-10, TNF*α*, IL-12p70, IL-1*β*, IL-6, and KC were observed in response to diabetes or NFAT inhibition; however plasma IFN-*γ* was reduced in diabetic animals that had been treated with A-285222 (Supplementary Table S1).

### 3.5. *In Vivo* Inhibition of NFAT Reduces Diabetes-Induced Vascular Permeability

**Figure 7:**
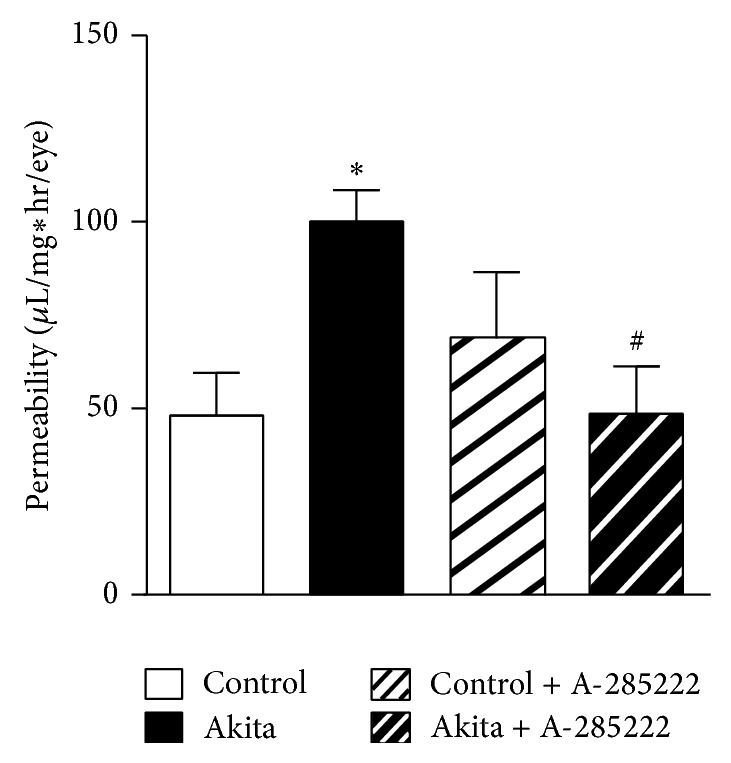
*In vivo* inhibition of NFAT reduces diabetes-induced vascular permeability. Vascular permeability (*μ*L/mg∗hr/eye) in the retinas of 8-week-old Akita mice and WT mice that had been treated for 2 weeks with daily i.p. injections of A-285222 (0.29 mg/kg) or saline (control). *N* = 8, 10, 7, and 7 for WT, Akita, WT + A-285222, and Akita + A-285222, respectively. Two-way ANOVA with Bonferroni post hoc test revealed ^∗^ *P* < 0.05 versus WT and ^##^ *P* < 0.05 versus Akita.

Vascular permeability was measured in the retinas of 8-week-old Akita and WT mice that had been treated with i.p. injections of A-285222 or saline (control) for 2 weeks. Mean blood glucose was 10.0 mmol/L and 18.5 mmol/L for control and Akita mice, respectively. Vascular permeability was increased 2.1-fold in diabetic versus control mice (Figure 7). Inhibition of NFAT signaling with A-285222 completely abrogated the diabetes-induced permeability (Figure 7).

## 4. Discussion 

In this study we show that mouse retinal vessels express NFATc3 and that this transcription factor is sensitive to changes in extracellular glucose levels. In two mouse models of diabetes, STZ-induced, and Akita mice, diabetes results in increased NFAT-dependent transcriptional activity in retinal vessels. Interestingly, levels of the potent anti-inflammatory cytokine IL-10 were decreased in the retina of diabetic mice and these were restored by treatment with the NFAT blocker A-285222 for 2 weeks. The effects on retinal IL-10 were achieved without any impact on blood glucose or on the levels of circulating plasma cytokines (IL-10, IFN-*γ*, IL-12p70, IL-1*β*, IL-6, KC, and TNF*α*). Moreover, inhibition of NFAT for 4 weeks resulted in decreased expression of basal* OPN* and* ICAM-1* mRNA levels in retinal vessels. We also demonstrate that inhibition of NFAT abrogates the increased vascular permeability observed in diabetic Akita mice. Therefore, we suggest that NFAT inhibition may exert a protective effect in the retina of diabetic mice.

The pattern of expression of* NFATc* transcription factors seems to vary depending on the vascular bed, with arteries such as the aorta that express all NFATc family members (Figure 1(c)), myometrial arteries expressing* NFATc1*,* NFATc3,* and* NFATc4* [7], or cerebral arteries which express* NFATc3* and* NFATc4* [24]. NFAT transcription factors were initially considered to have redundant functions, but differential activation of NFAT proteins within a cell [25] and varied expression profiles among cell types have been observed, suggesting functions specific to the different NFAT family members. In the intact mouse retina, all four isoforms could be detected, whereas in isolated retinal vessels only* NFATc2* and* NFATc3* were demonstrated. This suggests that NFATc1 and NFATc4 may be of neuronal origin [26, 27] or may be upregulated in culture, given that primary retinal endothelial cells expressed all four isoforms. Our previous work in conduit and resistance arteries focused on the effects of glucose on NFATc3 in vascular smooth muscle cells [2], but here we show that endothelial NFATc3 seems to be sensitive to changes in extracellular glucose as well. NFATc2, on the other hand, did not seem sensitive to glucose, at least in cells (Supplementary Figure 2).

Diabetes is characterized by both sustained hyperglycemia and acute glucose fluctuations. There is cogent evidence for the deleterious effects of sustained hyperglycemia, but the role of glucose variability is less well documented [28]. To our knowledge, nothing is known regarding the effect of glycemic peaks and nadirs on transcriptional activity in intact arteries. In mice, blood glucose reaches a maximum value upon a single IP-GTT after 30 minutes and returns to control levels after 120 minutes [29]. The results from this study show that acute elevations in blood glucose, as those induced by IP-GTT, efficiently induced NFAT nuclear accumulation and NFAT-dependent transcriptional activity in retinal vessels. Sustained hyperglycemia in STZ-treated mice resulted in increased NFAT transcriptional activity in retinal vessels. In contrast, NFAT transcriptional activity in whole retinas was not changed after an IP-GTT or after sustained hyperglycemia, suggesting different activation requirements of NFAT proteins in the nonvascular retina. Results also emphasize the importance of isolating retinal vessels rather than using whole-retina preparations for the study of pathological changes in the microvasculature.

The contribution of different cell types in the retina, as well as the chronology of events in the pathogenesis of DR, is still a matter of debate [6, 30, 31]. However, accumulating evidence suggests that localized inflammatory processes play a role in the early phases of DR [6], including increased expression of adhesion molecules, cytokines, and growth factors. For cytokine level quantification we chose a multiplex assay designed to measure IL-10, IFN-*γ*, IL-12p70, IL-1*β*, IL-6, KC, and TNF*α*. Except for KC, all of these cytokines have been implicated in the pathogenesis of human diabetic retinopathy [32–41]. In our study, 2 or 4 weeks of STZ-induced diabetes had no significant effect on the levels of circulating cytokines, despite the fact that animals had significantly higher blood glucose levels. The reason why this model fails to affect plasma cytokines as it could be predicted from previous studies may be due to differences between species. Rodents and in particular mice are known to be resilient to hyperglycemia-induced changes and only mimic early stages of diabetic retinopathy [42]. The plasma cytokine results presented here for FVBN and BALB/c mice are however in line with previous data obtained in C57BL/6, where we showed that 8 weeks of STZ-induced diabetes does not increase the levels of circulating inflammatory cytokines [43]. Treatment with A-285222 for 2 weeks had no effect on plasma cytokine levels, but 4-week treatment significantly reduced plasma IFN-*γ* concentration in diabetic animals (Table S1).

To better resolve potential local changes in cytokine production in the retina, we measured the same cytokines in retina homogenates. In the STZ-mouse model of diabetes that we used here, 2 weeks of diabetes resulted in decreased levels of IL-10. The same results were obtained after 4 weeks of diabetes in other strains (FVBN and BALB/c; data not shown). This cytokine is produced by numerous immune cells (i.e., T-helper cells, regulatory T cells, B cells, monocytes, macrophages, and dendritic cells [44]) and plays a central nonredundant role in limiting inflammation* in vivo* [45]. Also local production of endogenous IL-10 has been suggested to limit angiotensin-II-induced oxidative stress and vascular dysfunction in mouse carotid arteries [46]. Our results demonstrate that* in vivo* treatment for 2 weeks with A-285222 restores IL-10 levels, suggesting that NFAT inhibition may be protective for the retina. Apart from IL-10, no other changes in cytokine levels in retina or in the expression of* ICAM-1* mRNA in retinal vessels were observed in response to diabetes, at least at these early time points and in these mice strains. This contrasts with previous findings in C57Bl/6 mice at later time points after 8 weeks of diabetes, in which we were able to detect increased* ICAM-1* mRNA in retinal vessels and enhanced levels of* TNFα*,* IL-6,* and* IL-1β* mRNA in whole-retina homogenates [21].

We have recently shown that hyperglycemia induces the proinflammatory cytokine OPN in mouse aorta by direct binding of NFATc3 to the OPN promoter [3]. OPN protein was increased in subvalvular aortic sections and in plasma of STZ-treated diabetic mice 8 weeks after the onset of hyperglycemia [3]. OPN has been reported to be increased in the vitreous of patients with DR compared to nondiabetic patients [47] and in retinal endothelial cells after* in vitro* stimulation with high glucose, resulting in enhanced endothelial cell migration [48]. However, in the STZ-model used here, no changes in the expression of* OPN* in retinal vessels were observed at early time points, after 2 or 4 weeks of diabetes. Hyperglycemia in Akita mice also failed to induce* OPN* expression. In contrast, a 2.1-fold increase in retinal microvessel* OPN* mRNA was observed in hyperlipidemic* Apoe* ^−/−^ mice at 8 weeks after the first STZ injection, indicating that diabetes duration or metabolic state is of importance for regulation of OPN in retinal microvessels. However, despite the lack of diabetes-induced OPN expression in BALB/c mice after 4 weeks of diabetes, in agreement with previous results in larger vessels [3],* in vivo* NFAT-inhibition for the duration of the experiment reduced the levels of OPN mRNA in retinal vessels regardless of the diabetic condition, indicating a potential NFAT-dependent regulation of OPN under basal conditions in this tissue. NFAT inhibition also reduced* ICAM-1* mRNA levels in retinal microvessels, highlighting a potential mechanism by which NFAT inhibition may prevent leukostasis and inflammation in diabetic retinopathy.

An early feature of diabetic retinopathy is the breakdown of the BRB which results in vascular leakage and development of retinal edema. Clinical evidence from fluorescein angiography in patient with diabetic retinopathy indicates that the inner BRB, which is formed by junctions between endothelial cells of the retinal capillaries, is the primary site of vascular leakage. In agreement with previous studies [49], we demonstrate that increased vascular leakage is an early event in Akita mice. The fact that* in vivo* treatment with A-285222 for 2 weeks prevented diabetes-induced changes in retinal permeability suggests NFAT signaling to play a role in the regulation of the BRB. In diabetes, increased BRB permeability is associated with reduced expression of tight junction (claudin-5, ZO-1, and occludin) and adherens (VE-cadherin) proteins [50]. Interestingly, a high number of NFAT consensus binding sites were found in the promoter region of claudin-5 (26 sites), theoretically making it a possible direct target of NFAT regulation.

A-285222 is a low molecular weight (416 daltons) organic compound [51]. Given its lipophilic nature and small size, it crosses both the plasma membrane and the nuclear envelope by simple diffusion. We have previously studied the pharmacokinetics of A-285222 after i.p. administration in mice using gas chromatography/mass spectroscopy (GC/MS) [9]. A comparison between plasma levels of A-285222 measured 5 min after i.p. injection of the drug and after direct injection into the circulation (intracardiac) showed that levels were within the same range regardless of administration route, indicating high bioavailability and confirming transport across several membranes. Studies in cynomolgus monkeys also confirm the cell-permeable nature of the compound, as A-285222 was readily detected in plasma after oral administration [52]. As a cell-permeable compound A-285222 is expected to reach the retinal circulation and enter the cells in the vessel wall. The effects of treatment on vascular permeability could therefore be due to direct effects of A-285222 on vascular NFATc3 activation; however, indirect effects cannot be ruled out.

In conclusion, these results suggest that NFAT acts as a glucose-sensor in the wall of retinal microvessels, translating changes in extracellular glucose concentration into changes in gene expression leading to enhanced inflammation, endothelial activation, and vascular permeability. Even though tight blood glucose and blood pressure control are key in preventing and/or slowing down the development of diabetic macular edema and retinopathy, these are therapeutic goals difficult to achieve. Standard of care still relies on laser photocoagulation and intravitreal injections of corticosteroids or more recently anti-VEGF but these are invasive procedures that can have complications [53]. Therefore, new approaches beyond current standards of diabetes care are necessary. Here, we have identified the NFAT signaling as a putative target for treatment of microvascular complications in diabetes.

## Supplementary Material

Table S1. Plasma cytokines. Cytokine levels are expressed in ng/ml for OPN and in pg/ml for all other cytokines. Values represent mean ±SD. Plasma cytokine levels in control and STZ-treated BALB/c mice that have been treated with the NFAT inhibitor A-285222 (0.29 mg/kg for 2 weeks followed by 0.15 mg/kg for last 2 weeks) or saline, measured 4 weeks after the first STZ/vehicle injection. Interleukin (IL)-10, tumor necrosis factor (TNF)-α, interferon (IFN)-γ, IL-12p70, IL-1β, IL-6 and keratinocyte chemoattractant (KC) were measured using multiplex technology. n.d.; not detectable. Two-way ANOVA revealed no significant interactions, except for IFN-γ. For IFN-γ, one-way ANOVA and Bonferroni post-test revealed ∗P<0.05 vs STZ-treated saline group.
Supplementary Figure 1. Confocal immunofluorescence images showing staining of retinal whole-mounts with antibodies against von Willebrand factor (A; 1:400), smooth muscle α-actin (B; 1:400) and platelet-derived growth factor β-receptor (C; PDGFRβ, 1:100) for identification of endothelial, smooth muscle cells and pericytes, respectively. D. Smooth muscle α-actin and PDGFRβ double staining of retinal whole-mounts.
Supplementary Figure 2. Representative confocal immunofluorescence images of retinal whole mounts, stimulated for 30 min in high extracellular glucose (HG; 20 mmol/l) in the presence of A-285222 (A; 1μmol), or apyrase (B; 3.6 U/ml), or after stimulation with low D-glucose (LG; 2 mmol/l) plus mannitol (C; 18 mmol/l), or LG plus L-glucose (D; 18 mmol/l). Preparations were stained for NFATc3 (red) and SYTOX Green for identification of nuclei (green). Endothelial cells were identified by the orientation of their nuclei. Scale bars=50 μm.
Supplementary Figure 3. No significant effects of high glucose on NFATc2 nuclear accumulation in endothelial cells. A. Representative confocal immunofluorescence images of HUVEC stimulated for 30 min in low (LG; 2 mmol/l) or high (HG; 20 mmol/l; right panels) extracellular glucose with or without A-285222 (1μmol), stained for NFATc2 (red) and SYTOX Green for identification of nuclei (green). B. Summarized data from experiments as in (A), showing NFATc2 nuclear accumulation after 30 min stimulation in LG or HG in the presence or absence of A-285222 (1μmol), or after stimulation with LG plus mannitol (18 mmol/l). C. Summarized data from corresponding confocal experiments in HRMVECs, stimulated as in (A) and with VEGF (25 ng/ml) with or without A-285222 (1μmol).
Supplementary Figure 4. Expression of OPN mRNA in isolated retinal microvessels from normolipidemic (A-B) and dyslipidemic (C) diabetic mice, determined by quantitative RT-PCR. A. No differences between OPN mRNA expression levels in retinal vessels from diabetic and control NFAT-luc mice, measured 2 weeks after the first STZ/vehicle injection. HPRT was used as endogenous control. N=19 mice/group. B. No differences between OPN mRNA expression in retinal vessels from Akita and WT littermate control mice. 18S and Cyklophilin B were used as endogenous controls. N=12 and 4 for WT and Akita, respectively. C. OPN mRNA expression was significantly higher in diabetic Apoe^-/-^ mice when compared to non-diabetic Apoe^-/-^ mice measured 8 weeks after the first STZ/vehicle injection. HPRT and GAPDH were used as endogenous controls. N=11 mice/group, ^*^P<0.05.

## Figures and Tables

**Table 1:** A-285222 treatment does not affect plasma cytokine levels in diabetic NFAT-luc mice.

Cytokine (pg/mL)	Control (*N* = 10)	STZ (*N* = 13)	STZ + A-285222 (*N* = 8)
IL-10	22.0 ± 3.6	28.1 ± 7.0	29.4 ± 21.1
TNF-*α*	0.7 ± 0.3	0.6 ± 0.5	0.2 ± 0.5
IFN-*γ*	1.3 ± 1.3	1.2 ± 0.5	1.4 ± 0.8
IL-12p70	3.9 ± 5.5	8.2 ± 9.0	32.7 ± 56.2
IL-1*β*	1.0 ± 0.4	1.2 ± 0.5	1.1 ± 0.8
IL-6	13.3 ± 5.7	16.6 ± 5.8	25.0 ± 22.4
KC	51.4 ± 27.5	56.5 ± 17.7	64.1 ± 53.6

Data expressed as mean ± SD. Plasma cytokine levels (pg/mL) in control and STZ-treated NFAT-luc mice that had been treated with the NFAT inhibitor A-285222 (0.15 mg/kg/day) or saline, measured 2 weeks after the first STZ/vehicle injection. One-way ANOVA revealed no significant differences between groups. Interleukin- (IL-) 10, tumor necrosis factor- (TNF-) *α*, interferon- (IFN-) *γ*, IL-12p70, IL-1*β*, IL-6, and keratinocyte chemoattractant (KC) were measured using multiplex technology.
