# Supplementary material for: Nuclear Factor of Activated T Cells Is Activated in the Endothelium of Retinal Microvessels in Diabetic Mice
Source: J Diabetes Res. 2015 Mar 31;2015:428473. doi: 10.1155/2015/428473 (PMC4396720; doi:10.1155/2015/428473)
Supplement: Supplementary file 1 — Table S1. Plasma cytokines. Cytokine levels are expressed in ng/ml for OPN and in pg/ml for all other cytokines. Values represent mean ±SD. Plasma cytokine levels in control and STZ-treated BALB/c mice that have been treated with the NFAT inhibitor A-285222 (0.29 mg/kg for 2 weeks followed by 0.15 mg/kg for last 2 weeks) or saline, measured 4 weeks after the first STZ/vehicle injection. Interleukin (IL)-10, tumor necrosis factor (TNF)-α, interferon (IFN)-γ, IL-12p70, IL-1β, IL-6 and keratinocyte chemoattractant (KC) were measured using multiplex technology. n.d.; not detectable. Two-way ANOVA revealed no significant interactions, except for IFN-γ. For IFN-γ, one-way ANOVA and Bonferroni post-test revealed ∗P<0.05 vs STZ-treated saline group. Supplementary Figure 1. Confocal immunofluorescence images showing staining of retinal whole-mounts with antibodies against von Willebrand factor (A; 1:400), smooth muscle α-actin (B; 1:400) and platelet-derived growth factor β-receptor (C; PDGFRβ, 1:100) for identification of endothelial, smooth muscle cells and pericytes, respectively. D. Smooth muscle α-actin and PDGFRβ double staining of retinal whole-mounts. Supplementary Figure 2. Representative confocal immunofluorescence images of retinal whole mounts, stimulated for 30 min in high extracellular glucose (HG; 20 mmol/l) in the presence of A-285222 (A; 1μmol), or apyrase (B; 3.6 U/ml), or after stimulation with low D-glucose (LG; 2 mmol/l) plus mannitol (C; 18 mmol/l), or LG plus L-glucose (D; 18 mmol/l). Preparations were stained for NFATc3 (red) and SYTOX Green for identification of nuclei (green). Endothelial cells were identified by the orientation of their nuclei. Scale bars=50 μm. Supplementary Figure 3. No significant effects of high glucose on NFATc2 nuclear accumulation in endothelial cells. A. Representative confocal immunofluorescence images of HUVEC stimulated for 30 min in low (LG; 2 mmol/l) or high (HG; 20 mmol/l; right panels) extracellular glucose with or w [file 428473.f1.pdf]

Online Supplemental Material

Table S1. Plasma cytokines

Cytokine	Control (n=7)	STZ (n=8)	Control+A-285222 (n=8)	STZ+A-285222 (n=8)
OPN	81.0±11.4	94.2±32.5	76.5±12.1	84.9±20.7
IL-10	13.8±8.4	15.8±10.4	14.4±4.2	12.7±7.7
TNF-α	n.d.	0.2±0.7	0.2±0.4	n.d.
IFN-γ	1.9±0.9	3.4±3.3	2.1±1.0	1.0±0.6*
IL-12p70	6.7±8.9	8.7±8.5	4.8±7.8	16.6±27.6
IL-1β	0.6±0.3	1.1±0.8	0.8±0.2	0.7±0.4
IL-6	2.9±1.9	7.8±1.4	4.6±4.4	3.3±3.9
KC	39.0±5.6	60.6±41.5	68.2±17.1	68.1±27.5

Cytokine levels are expressed in ng/ml for OPN and in pg/ml for all other cytokines. Values represent mean ± SD. Plasma cytokine levels in control and STZ-treated BALB/c mice that have been treated with the NFAT inhibitor A-285222 (0.29 mg/kg for 2 weeks followed by 0.15 mg/kg for last 2 weeks) or saline, measured 4 weeks after the first STZ/vehicle injection. Interleukin (IL)-10, tumor necrosis factor (TNF)-α, interferon (IFN)-γ, IL-12p70, IL-1β, IL-6 and keratinocyte chemoattractant (KC) were measured using multiplex technology. n.d.; not detectable. Two-way ANOVA revealed no significant interactions, except for IFN-γ. For IFN-γ, one-way ANOVA and Bonferroni post-test revealed $^{*}P<0.05$ vs STZ-treated saline group.

Supplementary figure 1. Confocal immunofluorescence images showing staining of retinal whole-mounts with antibodies against von Willebrand factor (**A**; 1:400), smooth muscle α-actin (**B**; 1:400) and platelet-derived growth factor β-receptor (**C**; PDGFRβ, 1:100) for

identification of endothelial, smooth muscle cells and pericytes, respectively. **D**. Smooth muscle α-actin and PDGFRβ double staining of retinal whole-mounts.

Supplementary Figure 2. Representative confocal immunofluorescence images of retinal whole mounts, stimulated for 30 min in high extracellular glucose (HG; 20 mmol/l) in the presence of A-285222 (**A**; 1µmol), or apyrase (**B**; 3.6 U/ml), or after stimulation with low D-glucose (LG; 2 mmol/l) plus mannitol (**C**; 18 mmol/l), or LG plus L-glucose (**D**; 18 mmol/l). Preparations were stained for NFATc3 (red) and SYTOX Green for identification of nuclei (green). Endothelial cells were identified by the orientation of their nuclei. Scale bars=50 µm.

Supplementary Figure 3. No significant effects of high glucose on NFATc2 nuclear accumulation in endothelial cells. A. Representative confocal immunofluorescence images of HUVEC stimulated for 30 min in low (LG; 2 mmol/l) or high (HG; 20 mmol/l; right panels) extracellular glucose with or without A-285222 (1µmol), stained for NFATc2 (red) and SYTOX Green for identification of nuclei (green). **B.** Summarized data from experiments as in (A), showing NFATc2 nuclear accumulation after 30 min stimulation in LG or HG in the presence or absence of A-285222 (1µmol), or after stimulation with LG plus mannitol (18 mmol/l). **C**. Summarized data from corresponding confocal experiments in HRMVECs, stimulated as in (A) and with VEGF (25 ng/ml) with or without A-285222 (1µmol).

Supplementary Figure 4. Expression of *OPN* mRNA in isolated retinal microvessels from normolipidemic (A-B) and dyslipidemic (C) diabetic mice, determined by quantitative RT-PCR. **A**. No differences between *OPN* mRNA expression levels in retinal vessels from diabetic and control NFAT-luc mice, measured 2 weeks after the first STZ/vehicle injection. *HPRT* was used as endogenous control. N=19 mice/group. **B.** No differences between *OPN*

mRNA expression in retinal vessels from Akita and WT littermate control mice**.** *18S* and *Cyklophilin B* were used as endogenous controls. N=12 and 4 for WT and Akita, respectively. **C.** *OPN* mRNA expression was significantly higher in diabetic *Apoe*$^{-/-}$ mice when compared to non-diabetic *Apoe*$^{-/-}$ mice measured 8 weeks after the first STZ/vehicle injection. *HPRT* and *GAPDH* were used as endogenous controls. N=11 mice/group, $^{*}P<0.05$.

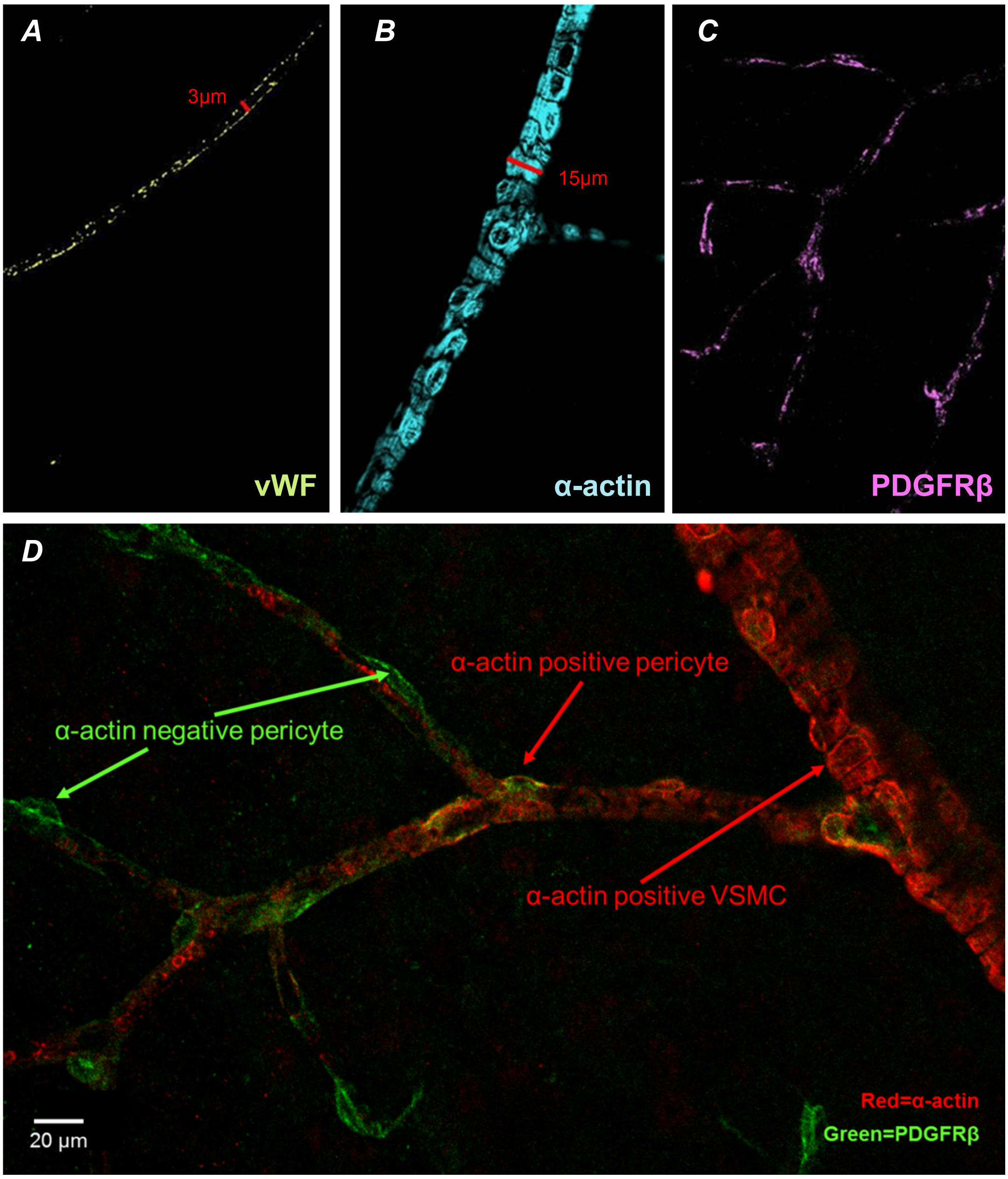

Supplemental figure 1

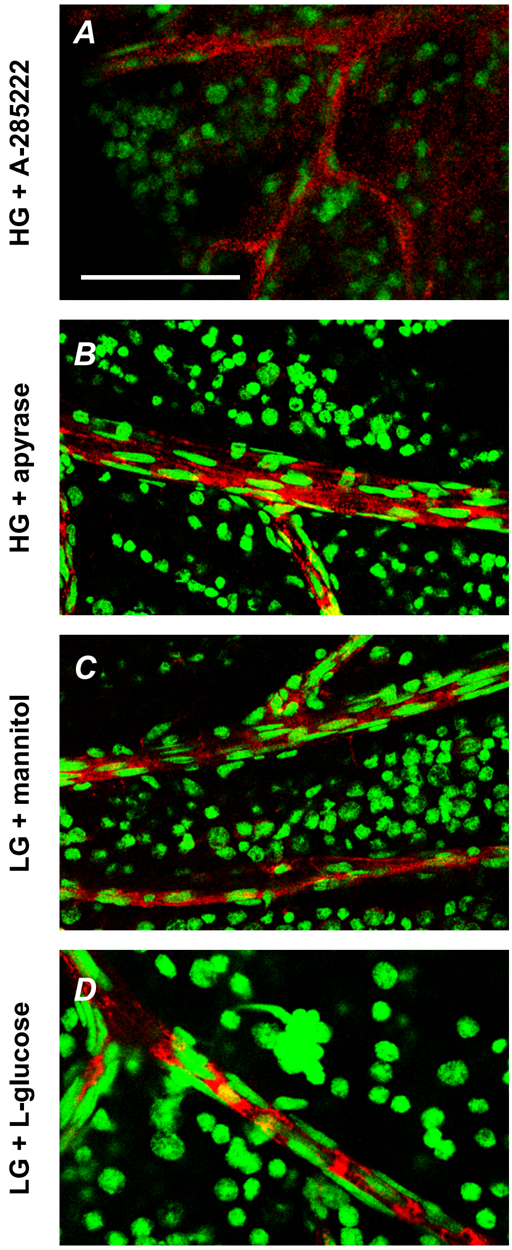

Supplemental figure 2

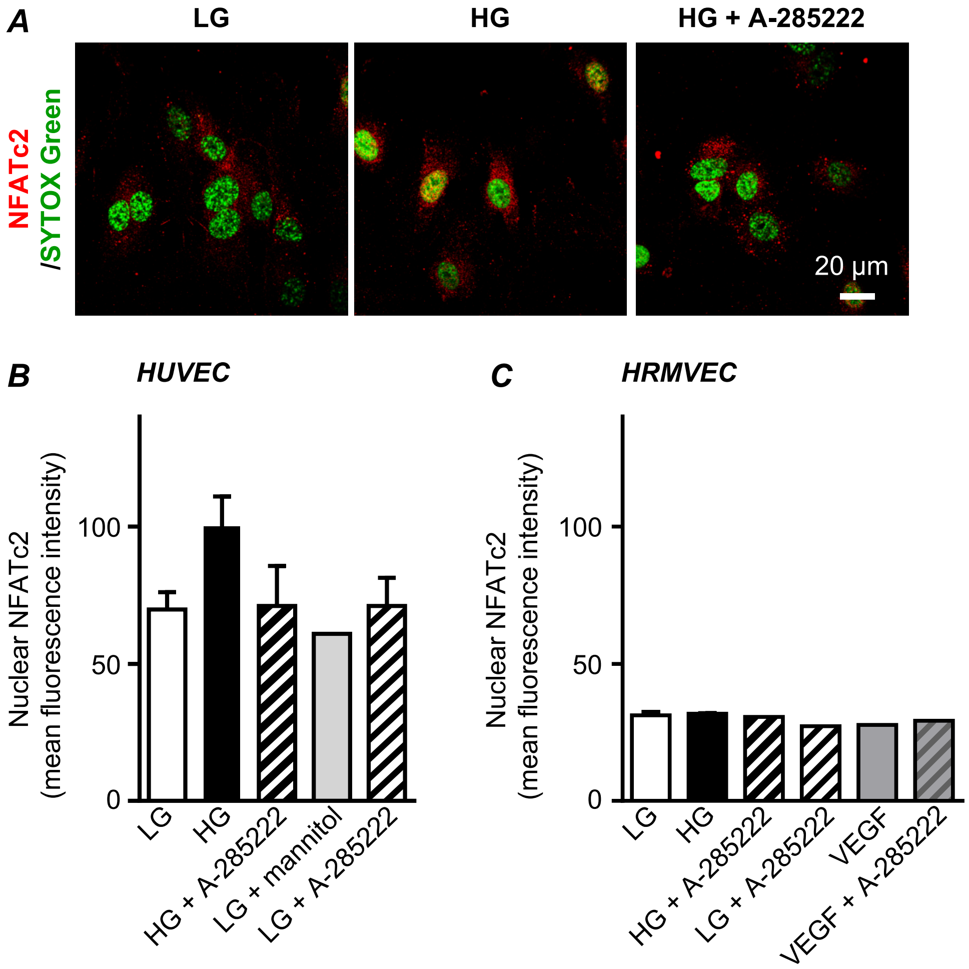

Supplemental figure 3

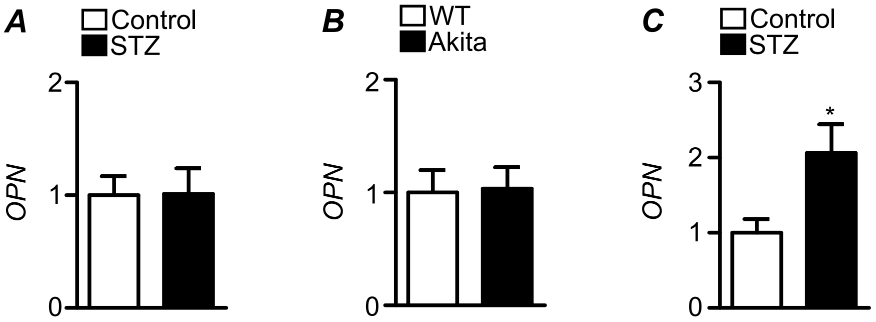

Supplemental figure 4
